# Measurable residual disease assessed by mass spectrometry in peripheral blood in multiple myeloma in a phase II trial of carfilzomib, lenalidomide, dexamethasone and autologous stem cell transplantation

**DOI:** 10.1038/s41408-021-00418-2

**Published:** 2021-02-05

**Authors:** Benjamin A. Derman, Andrew T. Stefka, Ken Jiang, Amanda McIver, Tadeusz Kubicki, Jagoda K. Jasielec, Andrzej J. Jakubowiak

**Affiliations:** 1 Section of Hematology/Oncology, University of Chicago Medical Center, Chicago, IL USA; 2 Department of Hematology and Bone Marrow Transplantation, Poznan University of Medical Sciences, Poznan, Poland

**Keywords:** Phase II trials, Myeloma

Dear Editor,

Despite advances in the detection of measurable (minimal) residual disease (MRD) in multiple myeloma (MM), some patients who are MRD negative by highly sensitive methods, such as multiparametric flow cytometry (MFC) and next-generation sequencing (NGS), still experience disease progression^1^. These bone marrow (BM)-based assessments may lead to false-negative results due to spatial heterogeneity in the BM, hemodilution, and/or extramedullary disease. Positron emission tomography/computed tomography (PET/CT) is complementary but gaps remain^2^.

Peripheral blood (PB)-based methods could potentially overcome these barriers in assessing for residual disease in MM, but time-tested methods such as serum protein electrophoresis (SPEP) and immunofixation (IFIX) that assess for the monoclonal immunoglobulin product in MM do not have sufficient sensitivity (LoD 0.1–0.2 g/dL with extended LoD 0.04 g/dL if migrate in the gamma fraction) as the monoclonal protein (MP) spike fades into the polyclonal background with lower levels of disease^3,4^. In contrast, mass spectrometry (MS) is able to detect the presence of a plasma cell clone at a heightened sensitivity via the mass/charge ratio of its secreted paraprotein, making it uniquely suited to assess for MRD^3,5–7^. Two prevailing techniques for MS have arisen for analyzing monoclonal immunoglobulins: matrix‐assisted laser desorption ionization time‐of‐flight MS (MALDI-TOF-MS)^6,8^ and liquid chromatography (LC) quadrupole time-of-flight MS^5,8^. While MALDI is a high-throughput method that allows for quick turnaround with LoD 0.05 g/dL, LC-MS can achieve higher sensitivity with LoD 0.005 g/dL.

In this study, we assessed the concordance and prognosticative abilities of residual disease status by MS in the PB compared with SPEP/IFIX, PET/CT, and MFC and NGS in the BM to determine if MS may represent a suitable PB-based method for detecting MRD in MM.

Seventy-six patients with newly diagnosed secretory MM were enrolled into a multicenter phase two study investigating the safety and efficacy of four cycles of carfilzomib, lenalidomide, and dexamethasone (KRd) induction followed by high-dose melphalan and autologous stem cell rescue, KRd consolidation for 14 cycles, and lenalidomide maintenance therapy (NCT01816971)^9^. Written informed consent was provided by each individual, and the study was approved by the local Institutional Review Board. Thirty-six patients (characteristics in Table S1) had paired PB and BM samples at the end of cycle 18 KRd (C18) and were included in this analysis; of these patients, 24 also had paired PB and BM samples available at the end of 1 year of lenalidomide maintenance (1yLM). All 36 patients reached a very good partial response or better, and 29 (81%) achieved a complete response or better. MRD by MFC was performed with an LoD between 10^−4^ and 10^−5^. MRD by NGS in the BM was performed using clonoSEQ (Adaptive Biotechnologies, Seattle, WA) prospectively per protocol with LoD <10^−5^ based on DNA input^10^. Of the 60 total NGS samples evaluated in this analysis, 18 reached an LoD <10^−6^, 40 reached an LoD <10^−5^, and 2 reached an LoD of 10^−4^–10^−5^.

MS was performed by the Binding Site Group using both MALDI-TOF-MS and LC-MS. Analysts performing the MS experiments were blinded to the status of each patient’s PB sample (MP isotype and concentration) prior to analysis. The MS methods used have been described previously^5,6^. The unique molecular mass of a monoclonal light chain was defined at baseline and was tracked in each patient’s samples^5^. MS results were only considered positive if the original paraprotein at diagnosis was detected.

Concordance between MS by MALDI-TOF and LC-MS in PB, IFIX in PB, PET/CT, MFC in BM, and NGS in BM were assessed using Cohen’s kappa (*κ*) statistic, with *κ* value interpretation per Landis and Koch^11^. Progression-free survival (PFS) curves were constructed using the Kaplan–Meier method and log-rank tests. Hazard ratios (HRs) and 95% confidence intervals (CIs) for survival data were derived from Cox proportional hazards regression models.

There was substantial concordance (*κ* = 0.667, 83% agreement) between NGS and MALDI-TOF-MS among the 60 samples (Fig. 1A, Fig. S1, and Table S2). Stratified by LoD of the NGS sample (Fig. S2A), the two NGS samples with LoD 10^−4^–10^−5^ were NGS^−^/MALDI-TOF^−^. There was 80% agreement (*κ* = 0.591) between MALDI-TOF-MS and NGS with LoD 10^−5^–10^−6^, and 89% agreement (*κ* = 0.766) between MALDI-TOF-MS and NGS with LoD <10^−6^. The only NGS^+^/MALDI-TOF-MS^−^ case was with an NGS sample with LoD <10^−6^, and 8/9 (78%) NGS^−^/MALDI-TOF-MS^+^ cases involved NGS samples with LoD between 10^−5^ and 10^−6^. Three (33%) of the NGS^−^/MALDI-TOF-MS^+^ cases clinically progressed, including one patient who converted to NGS^+^ at a later timepoint, but prior to clinical progression.

**Fig. 1:**
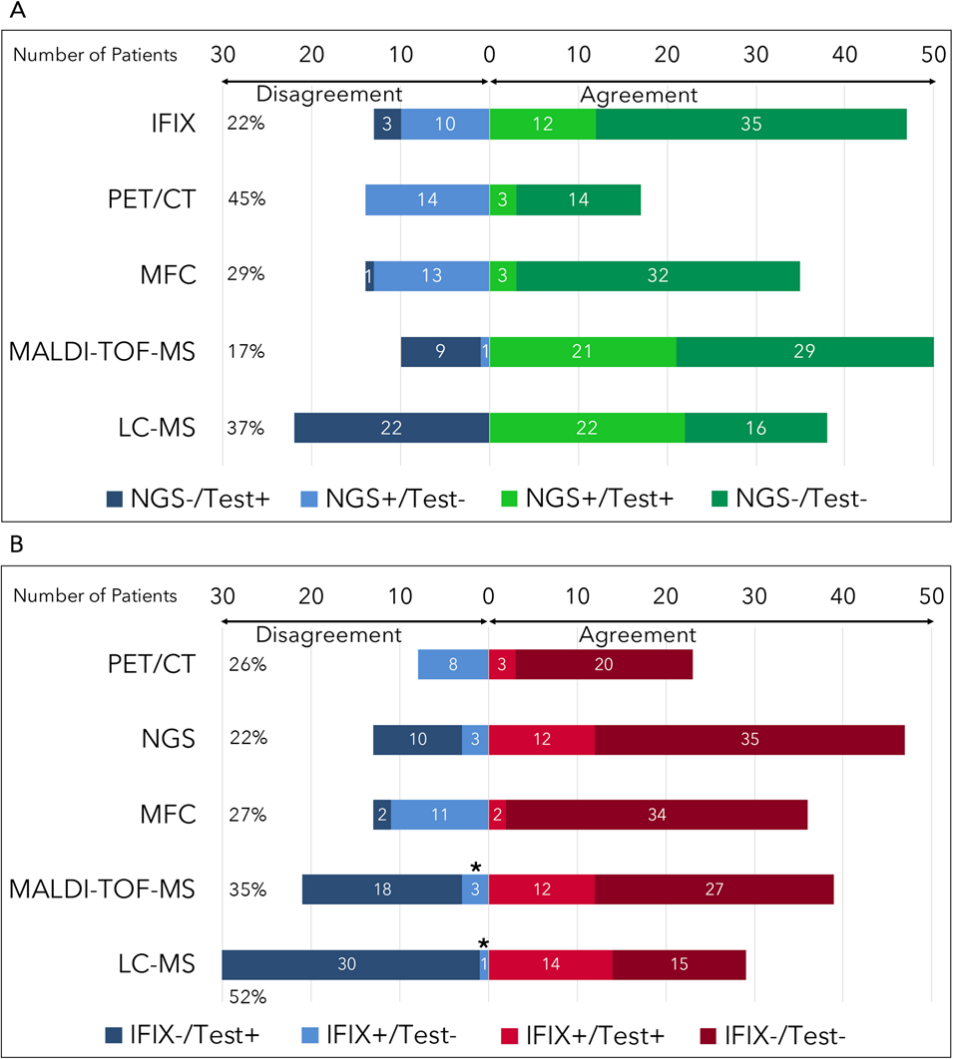
Dis/agreement among MRD testing methods. **A** Dis/agreement between next-generation sequencing (NGS) and other measurable residual disease (MRD) assays. **B** Dis/agreement between immunofixation (IFIX) and MRD assays. *Mass spectrometry (MS) identified a monoclonal protein of a different isotype from IFIX. LC-MS liquid chromatography mass spectrometry, MALDI-TOF-MS matrix‐assisted laser desorption ionization time‐of‐flight, MFC multiparameter flow cytometry, PET/CT positron emission tomography/computed tomography.

Concordance between NGS and LC-MS was fair (*κ* = 0.348) with 63% agreement (Table S3 and Fig. S2B). There was no NGS^+^/LC-MS^−^ case compared to 22 NGS^−^/LC-MS^+^ cases (from 16 unique patients over the two timepoints; Fig. 1A). Stratified by LoD of the NGS sample, the two NGS samples with LoD 10^−4^–10^−5^ were NGS^−^/LC-MS^+^. There was 60% agreement (*κ* = 0.309) between LC-MS and NGS with LoD between 10^−5^ and 10^−6^; 16/40 (40%) samples were NGS^−^/LC-MS^+^. There was 78% agreement (*κ* = 0.478) between LC-MS and NGS with an LoD <10^−6^ with 4/18 (22%) samples being NGS^−^/LC-MS^+^ (Fig. S2B). Of the 16 total patients with discordant NGS^−^/LC-MS^+^ cases at C18, 5 (31%) experienced progression, including 3 who converted to NGS^+^ at a later timepoint, but prior to the clinical progression^12^. An example of MALDI-TOF and LC-MS mass spectra of a patient with NGS^−^/MALDI-TOF-MS^−^/LC-MS^+^ status at C18 is shown in Fig. S3.

A MP was identified at diagnosis in all 36 patients by both IFIX and MALDI-TOF-MS/LC-MS. There were only three patients who were IFIX^+^/MS^−^(Fig. 1B and Fig. S1); all of these patients were found to have a different clone of the same isotype by MS (i.e., an IgG kappa paraprotein of an entirely different molecular mass from the original), suggesting that IFIX^+^ results in these patients did not correspond with the original clone at diagnosis.

MFC (LoD 10^−4^–10^−5^) and PET/CT results were available at the same timepoints in 49/60 (82%) and 31/60 (52%) samples, respectively. Neither MFC nor PET/CT offered additional discriminatory capacity (Fig. 1 and Fig. S1).

PFS by MRD status at C18 is shown in Fig. 2. With a median follow-up of 56 months, MRD negativity by NGS was associated with a nonsignificant PFS benefit (HR 2.9, 95% CI 0.8–10.8, *p* = 0.11), as was the case for MALDI-TOF-MS negativity (HR 2.5, 95% CI 0.6–9.7, *p* = 0.17). Of the nine patients who were LC-MS^−^ at C18, all were alive and free of progression at last follow-up compared to the ten events (including four deaths) in the C18 LC-MS^+^ group (log-rank *p* = 0.026). When limiting analysis to only the patients who were MRD negative by NGS, NGS^−^/LC-MS^−^ (*n* = 9) was associated with superior PFS compared to NGS^−^/LC-MS^+^ (*n* = 14, log-rank *p* = 0.04). Comparison of PFS between NGS^−^/MALDI-TOF-MS^−^ (*n* = 16) and NGS^−^/MALDI-TOF-MS^+^ (*n* = 7) was limited by a wide CI, though the effect size appeared clinically meaningful (HR 3.4, 95% CI 0.6–20.1, *p* = 0.2).

**Fig. 2:**
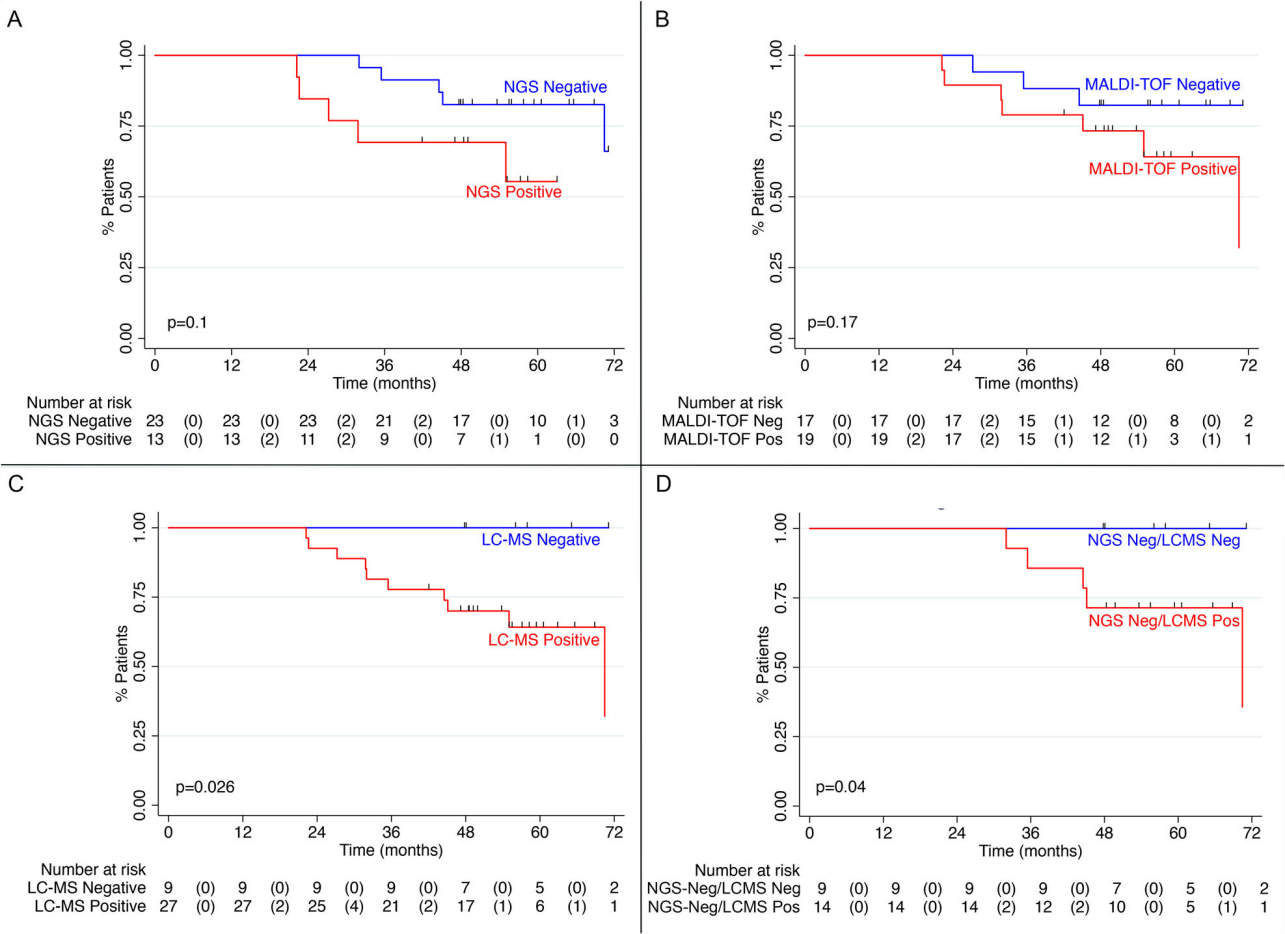
Progression-free survival by MRD status after 18 cycles KRd. **A** PFS by NGS status after 18 cycles of KRd; **B** PFS by MALDI-TOF-MS status after 18 cycles of KRd; **C** PFS by LC-MS status after 18 cycles; **D** PFS of all NGS^−^ patients, stratified by LC-MS status. Numbers in parentheses indicate events. KRd carfilzomib, lenalidomide, dexamethasone, LC-MS liquid chromatography mass spectrometry, MALDI-TOF-MS matrix‐assisted laser desorption ionization time‐of‐flight, MRD measurable residual disease, NGS next-generation sequencing; PFS progression-free survival.

Prior studies have suggested that assessments for malignant plasma cell clones by NGS and next-generation flow (NGF) in the PB have not been able to approximate the LoD of BM testing^13,14^. This is the first study to show that a MS-based PB “liquid biopsy” method for residual disease may be at least as sensitive as BM-based MRD assessments designed to detect one malignant cell per million nucleated cells (LoD ≤ 10^−6^) and possibly more sensitive than the current standard of LoD < 10^−5^_,_ suggesting that MS could be considered for MRD detection.

We found that MRD assessment by MALDI-TOF-MS in the PB was at least as sensitive as MRD by NGS with LoD < 10^−5^ in the BM, while LC-MS in the PB was superior to the current standard of MRD negativity with LoD < 10^−5^ and at least as sensitive as MRD by NGS with LoD < 10^−6^ in the BM. Moreover, LC-MS status appeared to be a superior predictor of PFS compared to NGS or MALDI-TOF-MS status; none of the patients who were LC-MS^−^ at C18 experienced progression or death. That a fraction of LC-MS^+^/NGS^−^ patients later converted to NGS^+^ and experienced clinical progression suggests LC-MS is a highly sensitive PB technique for detection of MRD that may predict progression sooner than state-of-the-art BM-based assays. It is unlikely that the MS^+^ cases in this study represented false positives as even the earliest PB samples analyzed were nearly 2 years from the start of treatment, when recirculation of existing MP is not expected.

Current evidence suggests that MS in the PB and NGF/NGS in the BM are complementary methods whose results should be considered as part of a multimodal approach to MRD assessment^15^. Though its use is rising, MS as an assay for MRD is limited to the research setting at the moment, owing to lack of consensus on the appropriate technique (MALDI-TOF-MS vs LC-MS) and availability. It will also be important to factor the cost of these methods into a disease monitoring algorithm. If validated, MS in the PB could be used as a screening method for MRD for patients who have undetectable disease by IFIX and serum free light chains; if MS is negative for disease, a BM biopsy and aspiration could be performed to confirm MRD-negative status. Comprehensive multimodal MRD approaches that incorporate functional imaging with assessments of the BM and PB compartments may allow for improved MRD-adaptive clinical trials, and allow for MRD to better guide decision-making in MM.

## Supplementary information

Supplemental Appendix

Checklist for NPG

## References

[1] Perrot A (2018). Minimal residual disease negativity using deep sequencing is a major prognostic factor in multiple myeloma. Blood.

[2] Moreau P (2017). Prospective evaluation of magnetic resonance imaging and [18F]fluorodeoxyglucose positron emission tomography-computed tomography at diagnosis and before maintenance therapy in symptomatic patients with multiple myeloma included in the IFM/DFCI 2009 trial: results of the IMAJEM study. J. Clin. Oncol..

[3] Zajec M (2020). Mass spectrometry for identification, monitoring, and minimal residual disease detection of M-proteins. Clin. Chem..

[4] Murray DL (2012). Laboratory persistence and clinical progression of small monoclonal abnormalities. Am. J. Clin. Pathol..

[5] Barnidge DR (2014). Using mass spectrometry to monitor monoclonal immunoglobulins in patients with a monoclonal gammopathy. J. Proteome Res..

[6] Mills JR (2016). Comprehensive assessment of M-proteins using nanobody enrichment coupled to MALDI-TOF mass spectrometry. Clin. Chem..

[7] Mills JR, Barnidge DR, Dispenzieri A, Murray DL (2017). High sensitivity blood-based M-protein detection in sCR patients with multiple myeloma. Blood Cancer J..

[8] Chapman JR, Thoren KL (2020). Tracking of low disease burden in multiple myeloma: Using mass spectrometry assays in peripheral blood. Best Pract. Res. Clin. Haematol..

[9] Jasielec, J et al. Carfilzomib, lenalidomide, and dexamethasone plus transplant in newly diagnosed multiple myeloma. *Blood* **136**, 2513–2523 (2020). 10.1182/blood.2020007522 PMC7714092 32735641

[10] Adaptive Biotechnologies Corporation. clonoSEQ Assay Technical Information. https://www.clonoseq.com/sites/default/files/clonoSEQ_TechnicalInformationSummary_21Sept2018.pdf (2010).

[11] Landis JR, Koch GG (1977). The measurement of observer agreement for categorical data. Biometrics.

[12] Kumar S (2016). International Myeloma Working Group consensus criteria for response and minimal residual disease assessment in multiple myeloma. Lancet Oncol..

[13] Vij R (2014). Deep sequencing reveals myeloma cells in peripheral blood in majority of multiple myeloma patients. Clin. Lymphoma Myeloma Leuk..

[14] Sanoja-Flores L (2019). Blood monitoring of circulating tumor plasma cells by next generation flow in multiple myeloma after therapy. Blood.

[15] Puíg N (2020). Analysis of treatment efficacy in the GEM-CESAR trial for high-risk smoldering multiple myeloma patients: comparison between the standard and IMWG MRD criteria and QIP-MS including FLC (QIP-FLC-MS). J. Clin. Oncol..

