# Supplementary material for: Measurable residual disease assessed by mass spectrometry in peripheral blood in multiple myeloma in a phase II trial of carfilzomib, lenalidomide, dexamethasone and autologous stem cell transplantation
Source: Blood Cancer J. 2021 Feb 5;11(2):19. doi: 10.1038/s41408-021-00418-2 (PMC7873068; doi:10.1038/s41408-021-00418-2)
Supplement: Supplementary file 1 — Supplemental Appendix [file 41408_2021_418_MOESM1_ESM.pdf]

Supplement

Measurable Residual Disease Assessed by Mass Spectrometry in Peripheral Blood in Multiple Myeloma in a Phase II Trial of Carfilzomib, Lenalidomide, Dexamethasone and Autologous Stem Cell Transplantation

Supplementary Appendix

PAGE

Table S1: **Patient Characteristics……………………………………………….….…..…...2**
Table S2: **Concordance Between MALDI-TOF-MS and NGS……………………….….…..3**
Table S3: **Concordance Between LC-MS and NGS……………………………….…….....3**
Figure S1: **Concordance Between Tests for Measurable Residual Disease……………….4**
Figure S2: **Dis/Agreement Between MS and NGS Stratified by Limit of Detection…...…5**
Figure S3: **MALDI-TOF and LC Light Chain Mass Spectra……………………………...6**

Supplement

Table S1: Patient Characteristics

Characteristic	Patients (n=36)
**Age, median (range)**	55 (40-72)
≥ 65 years, n (%)	7 (19%)
**Gender**	
Male	16 (44%)
**Race/Ethnicity**	
White/Non-Hispanic	26 (72%)
African Ancestry	5 (14%)
Hispanic	4 (11%)
Asian	1 (3%)
**International Staging System**	
1	17 (47%)
2	15 (42%)
3	4 (11%)
**High-Risk Cytogenetic Abnormality Present***	12 (33%)
**# of Cycles of KRd Received, median (range)**	18 (11-18)
# Received < 18 Cycles	6 (17%)
**Response After Cycle 18 KRd**	
VGPR/nCR	8 (22%)
≥ CR	28 (78%)

*Defined as: t(4;14), del(17p), t(14;16), t(14;20), and/or gain(1q).

Abbreviations: CR = complete response; KRd = Carfilzomib, Lenalidomide, Dexamethasone; nCR = near complete response; VGPR = very good partial response

Table S2: Concordance Between MALDI-TOF-MS and NGS

Test Result		End of Cycle 18 KRd (n=36)	End of 1 Year of Maintenance (n=24)	All cases (n=60)
**NGS**	**MALDI-TOF-MS**			
(+)	(+)	12	9	21
(–)	(–)	16	13	29
(+)	(–)	1	0	1
(–)	(+)	7	2	9
**Kappa Statistic (95% CI)**		0.562 (0.309-0.815)	0.830 (0.607-1.0)	0.667 (0.485-0.848)
**% Agreement**		78%	92%	83%

Abbreviations: CI = confidence interval; KRd = Carfilzomib, Lenalidomide, Dexamethasone; MALDI-TOF-MS = matrix-assisted laser desorption ionization time-of-flight mass spectrometry; NGS = next generation sequencing.

Table S3: Concordance Between LC-MS and NGS

Test Result		End of Cycle 18 KRd (n=36)	End of 1 Year of Maintenance (n=24)	All Cases (n=60)
**NGS**	**LC-MS**			
(+)	(+)	13	9	22
(–)	(–)	9	7	16
(+)	(–)	0	0	0
(–)	(+)	14	8	22*
**Kappa Statistic (95% CI)**		0.317 (0.113-0.521)	0.396 (0.124-0.669)	0.348 (0.183-0.512)
**% Agreement**		61%	67%	63%

*22 cases for 16 unique patients

Abbreviations: CI = confidence interval; KRd = Carfilzomib, Lenalidomide, Dexamethasone; LC-MS = liquid chromatography mass spectrometry; NGS = next generation sequencing.

Supplement

Figure S1

	PET/CT	MFC	NGS	MALDI-TOF-MS	LC-MS
IFIX	0.326	0.126	0.500	0.300	0.162
PET/CT		0.625	0.162	0.111	0.05
MFC			0.195	0.086	0.069
NGS				0.667	0.348
MALDI-TOF-MS					0.533

κ = 0.0-0.2 Slight	κ = 0.21-0.4 Fair	κ = 0.41-0.6 Moderate	κ = 0.61-0.8 Substantial

Concordance Between Tests for Measurable Residual Disease.
Concordance was assessed using the kappa (κ) statistic. **Abbreviations:** IFIX = immunofixation; LC-MS = Liquid chromatography mass spectrometry; MALDI-TOF-MS = matrix-assisted laser desorption ionization time-of-flight; MFC = multiparameter flow cytometry; NGS = next generation sequencing.

Supplement

Figure S2

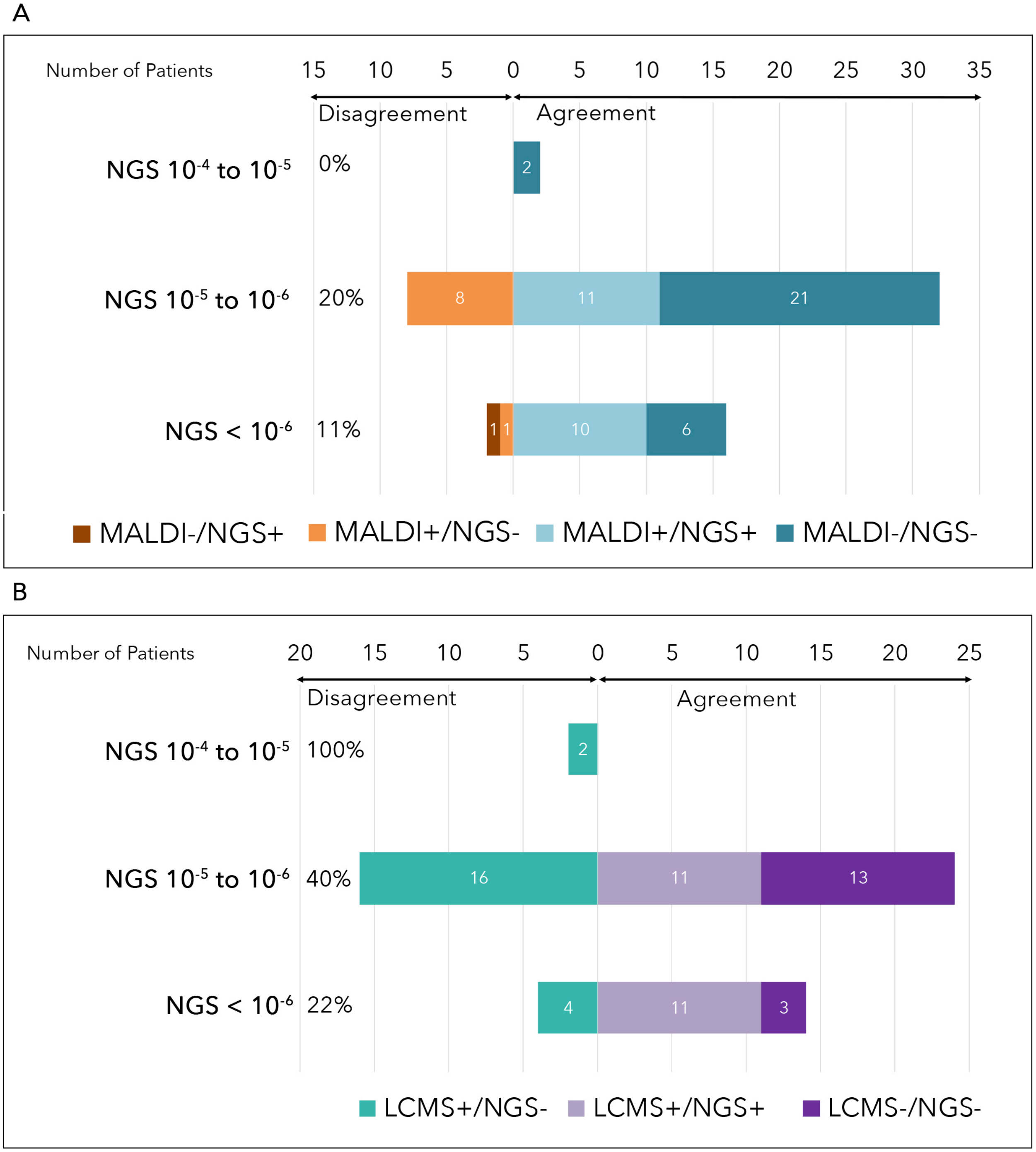

Dis/Agreement Between (A) MALDI-TOF-MS and NGS MRD methods and (B) LC-MS and NGS MRD methods stratified by the limit of detection for NGS.
Abbreviations: LCMS = Liquid chromatography mass spectrometry; MALDI = matrix-assisted laser desorption ionization time-of-flight; NGS = next generation sequencing.

Supplement

Figure S3

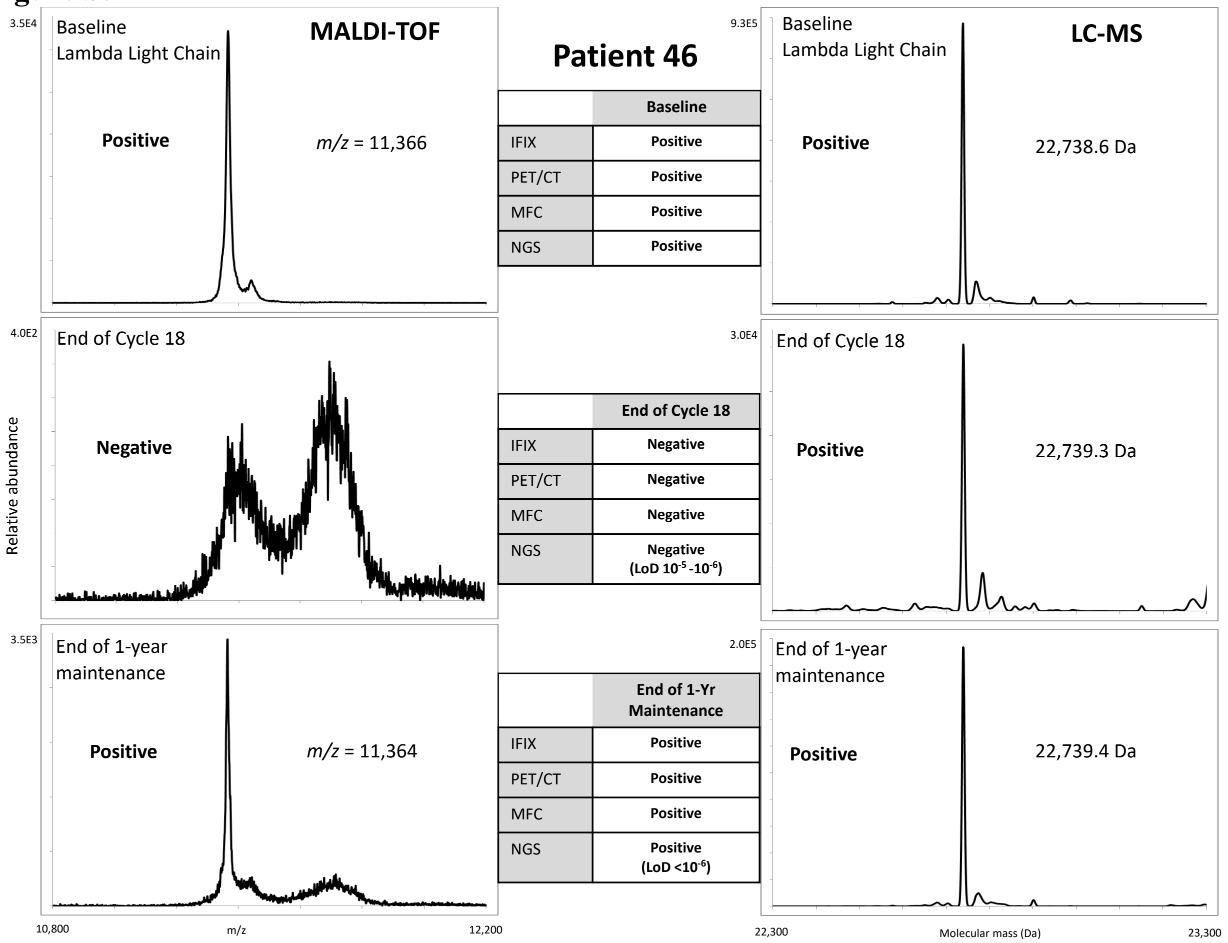

MALDI-TOF (left) and LC (right) light chain mass spectra shown for an individual patient, along with corresponding immunofixation (IFIX), positron emission tomography (PET), multiparameter flow cytometry (MFC), and next generation sequencing (NGS) results. The patient had an IgG Lambda paraprotein at diagnosis. The MALDI-TOF mass spectra show the +2 charge state of the Lambda light chain as the mass to charge ratio (m/z). The LC-MS mass spectra are deconvoluted spectra derived from electrospray ionization mass spectra and show the accurate molecular mass of the Lambda light chain in Daltons (Da). LoD = Limit of detection.
